# Benefits of an automated postoperative delirium risk prediction tool combined with non-pharmacological delirium prevention on delirium incidence and length of stay: a before–after analysis based on a quality improvement project

**DOI:** 10.1093/ageing/afae219

**Published:** 2024-10-14

**Authors:** Benjamin T Dodsworth, Kelly A Reeve, Martin Zozman, Philipp Meier, Felix Buddeberg, Marius Möller, Simone Pascale Wildhaber, Mary-Anne Kedda, Sönke Böttger, Reto Stocker, Nayeli Schmutz Gelsomino

**Affiliations:** PIPRA AG, Zurich, Switzerland; PIPRA AG, Zurich, Switzerland; Institute of Data Analysis and Process Design, Zurich University of Applied Sciences, Winterthur, Switzerland; NEXUS Personalized Health Technologies, ETH Zurich, Zurich, Switzerland; Kusnacht Practice AG, Nursing and Clinical Quality, Zollikon, Zurich, Switzerland; Klinik Hirslanden, Quality Management, Zurich, Switzerland; Klinik Hirslanden, Quality Management, Zurich, Switzerland; Klinik Hirslanden, Quality Management, Zurich, Switzerland; Klinik Hirslanden, Quality Management, Zurich, Switzerland; PIPRA AG, Zurich, Switzerland; Klinik Hirslanden, Quality Management, Zurich, Switzerland; Stiftung Quality of Life, Zurich, Switzerland; PIPRA AG, Zurich, Switzerland; Department of Clinical Research, University of Basel, Basel, Switzerland

**Keywords:** postoperative, delirium, non-pharmacological prevention, length of stay, risk, older people

## Abstract

**Background:**

Postoperative delirium (POD) significantly impacts older surgical patients, necessitating effective prevention strategies.

**Objective:**

To assess the effectiveness of the Pre-Interventional Preventive Risk Assessment (PIPRA) automated delirium risk prediction tool alongside non-pharmacological prevention strategies on POD incidence, hospital length of stay (LOS) and nursing time.

**Methods:**

This quality improvement project, set in a 335-bed Swiss private hospital, employed a before–after design to evaluate the impact of PIPRA and preventive measures on POD, LOS and nursing time in non-cardiac and non-intracranial surgery inpatients aged 60 or older. The control phase focused on enhancing POD screening, whilst the intervention phase incorporated PIPRA for risk assessment and staff training to enable targeted non-pharmacological prevention in patients at risk.

**Results:**

A total of 866 patients were included; 299 control and 567 intervention. The odds ratio of POD, comparing the intervention group to the control, was 0.71 [95% confidence interval (CI) 0.44–1.16] when adjusting for baseline patient characteristics. The intervention was associated with an LOS 0.94 (95% CI 0.85–1.05) and nursing time 0.96 (95% CI 0.86–1.07) times that of the control, adjusted for baseline patient characteristics. Medium risk patients (21.6% of patients) had an LOS 0.74 (95% CI 0.59–0.92) and required nursing time 0.79 (95% CI from 0.62–1.00) times the control, adjusted for baseline patient characteristics, equivalent to an LOS reduction of 1.36 days and nursing time saving of 19.3 hours per patient.

**Conclusions:**

Medium risk patients in the intervention group had shorter LOS and nursing time compared to the control group, underscoring the importance of targeted prevention.

## Graphical Abstract

**Graphical Abstract:**
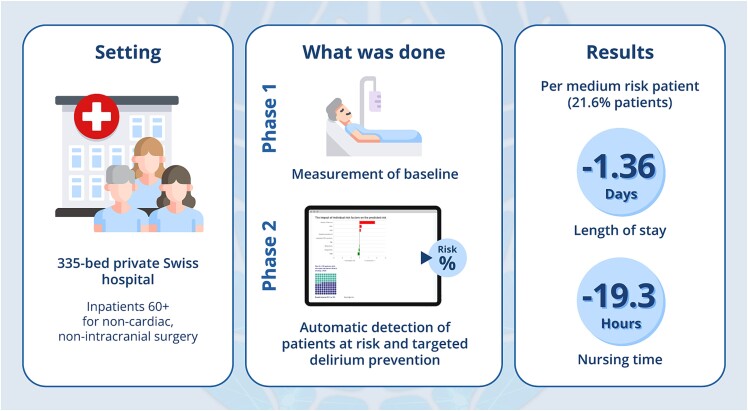

## Key Points

- Companion article to *Cost effectiveness of adopting a postoperative delirium risk prediction tool with non-pharmacological delirium prevention interventions for surgical patients* [1]
- Non-pharmacological delirium prevention performed on patients at risk
- Medium risk patients benefit from delirium prevention
- The Pre-Interventional Preventive Risk Assessment (PIPRA) tool supports targeted delirium prevention

## Introduction

Postoperative delirium (POD) is a common and serious complication of surgery [2], affecting up to 50% of patients over 65 years of age [3, 4]. POD is characterised by confusion, agitation and altered consciousness, and is associated with prolonged length of stay (LOS), higher readmission rates, functional decline, postoperative neurocognitive disorders (PND), dementia and increased mortality [5–13]. International medical societies strongly recommend that health care providers identify patients at risk for POD [2, 14, 15].

Efforts have been made to reduce POD incidence, with non-pharmacological multicomponent interventions [15] showing promise in preventing up to 54% of delirium cases [16]. However, there is still a substantial knowledge gap and these interventions are not routinely implemented by all healthcare providers. Furthermore, up to 70% of delirium cases go undiagnosed in hospitals [17], due to limited healthcare professional availability [18], complex aetiology and incomplete POD screening criteria.

Pre-Interventional Preventive Risk Assessment (PIPRA) is an automated, Conformité Européene (CE)-certified POD risk prediction tool developed [19, 20] for clinicians, to identify at risk patients over 60 years of age, thereby enabling implementation of targeted preventive strategies to reduce the incidence of POD.

The aim of this study was to implement and assess the effectiveness of PIPRA, combined with non-pharmacological delirium prevention, on delirium incidence, LOS and nursing time in surgical inpatients aged 60 or older, excluding cardiac and intracranial surgeries. We also investigated the association between the delirium risk and LOS/nursing time. Whilst there is compelling evidence that links delirium to increased LOS, the relationship between delirium risk and LOS or nursing time has, to the best of our knowledge, not yet been investigated.

## Methods

### Context

To assess the effect of implementing PIPRA, together with non-pharmacological prevention, we designed a quality improvement project (QIP) using a before–after study design. The QIP was performed in a 335-bed Swiss private hospital. In 2023/24 the hospital had a mix of 43.0% public and 57.0% private or partially privately insured patients, 79.4% of patients were outpatients and, of the inpatients, 82.4% were elective. The site was chosen as it was planning to introduce PIPRA. The project included all eligible patients admitted to the hospital from 1 May 2023 to 30 June 2023. Patients were eligible if they were surgical inpatients (excluding cardiac and intra-cranial surgery) aged 60 years or older.

The study comprised two phases. The before phase, Phase 1 ‘control’ (1 May to 22 May), was dedicated to improving POD screening and treatment. A staff education drive was conducted to increase POD awareness and the importance of regular assessment. Ward nurses were instructed to systematically screen for POD using the delirium observation screening scale (DOSS) [21] once per shift per patient, equivalent to three times per day. We also trained staff to treat delirium during Phase 1. The after phase, Phase 2 (23 May to 30 June), was dedicated to risk prediction and POD prevention. In this phase, staff were additionally instructed to apply the PIPRA tool to assess patient risk and to perform preventive measures on those at risk. The combination of risk prediction using PIPRA and targeted preventive measures was considered the study ‘intervention’. Patients were allocated to phases according to their admission date and all data was collected prospectively. The null hypothesis was that there would be no difference in delirium incidence or LOS between the phases.

Hospital nurses performed the delirium screening, treatment and prevention. A delirium team, with a consultant psychiatrist and internal medicine specialists, became operational 4 months before the start of the QIP and was available to support the nurses.

### Interventions

#### Training

Before the project started, we trained the head nurses of each of 14 wards over several weeks. We then supplied the head nurses with presentation materials to train their ward nurses, with instructions on delirium in general, and on the early identification and care of delirium. We provided leaflets describing how to correctly fill out the DOSS, and information brochures to give to relatives and sitters of delirious patients. These were accompanied by a hospital-wide awareness campaign (Delirium Awareness Month) that targeted all stakeholders (doctors, nurses, staff), and included weekly newsletters, informational videos and posters in break rooms.

Prior to the start of Phase 2, we educated the head nurses on the prevention of delirium and provided additional learning materials. The nurses were instructed to perform prevention strategies on patients with at least medium risk (≥10%) of developing POD. We also designed and distributed a pocket guide for the nurses, outlining all the steps for prevention, and we visited the nurses on the wards regularly, to help them identify any problems.

The bundle of non-pharmacological interventions for preventing delirium included thorough patient history-taking upon admission, encouragement of family involvement, empathetic communication, daily assessment of catheter needs (urinary, intravenous), early and regular mobilisation, multimodal pain management and enhancement of sleep–wake patterns (exposure to daylight and avoidance of naps during daytime, and minimising light, noise and patient care during the night). The nurses were also asked to orient the patients regularly, through communication and provision of pictures of relatives, personal items, hearing aids, glasses and to communicate a structured daily schedule, supported by the provision of large clocks, calendars and whiteboards.

#### Automatic identification of delirium risk

PIPRA is an automated delirium risk prediction tool, based upon a machine-learning algorithm developed from data from several clinical studies conducted around the world [20]. It estimates the risk, or probability, of an individual developing POD based upon routine patient and procedure information collected prior to surgery. The tool notifies nurses and any treating physicians if a patient is at increased risk by providing a notification in the electronic health record. In the study hospital, it was also configured to send a daily email to the delirium team, summarising all patients at risk and those who had been identified as delirious by the DOSS screening.

#### Measures

The primary outcome was delirium incidence. Patients were classified as delirious if they had at least one DOSS value of 3 or above during their hospital stay or had a formal clinical diagnosis of delirium. The DOSS [21] was chosen as it is a simple screening tool that does not require interaction with the patient. The expected incidence of delirium was calculated as the average delirium risk, according to PIPRA score, of all eligible patients admitted during a phase.

The secondary outcome was LOS, which was the time between a patient’s admission and discharge from hospital, chosen because increased LOS is known to be associated with delirium, mortality and readmission [22], and can be objectively measured, regardless of screening compliance. Compliance with delirium screening was also measured and was defined as number of actual DOSS screenings performed each day divided by the expected number of DOSS screenings per day, where the latter was three DOSS values daily for up to 5 days after surgery or discharge, whichever occurred first.

An exploratory outcome was the estimated time spent by the nurses as captured by ``Leistungserfassung in der Pflege'' (LEP). This system estimates the time spent by the nurses on a given patient based on which nursing interventions were performed and how long these take on average [23]. This was added since delirium prevention requires nursing time but successful delirium prevention also saves time.

#### Methods to ensure completeness of data

Following measurement of DOSS screening compliance, an automated daily email was sent to all head nurses, showing compliance at the ward-level.

For delirium risk assessment, the variables age, body mass index, American Society of Anesthesiologists score, number of prescribed medications, cognitive impairment, history of delirium, surgery risk, preoperative c-reactive protein (optional) and laparotomy/thoracotomy were routinely collected.

#### Analysis

**Figure 1:**
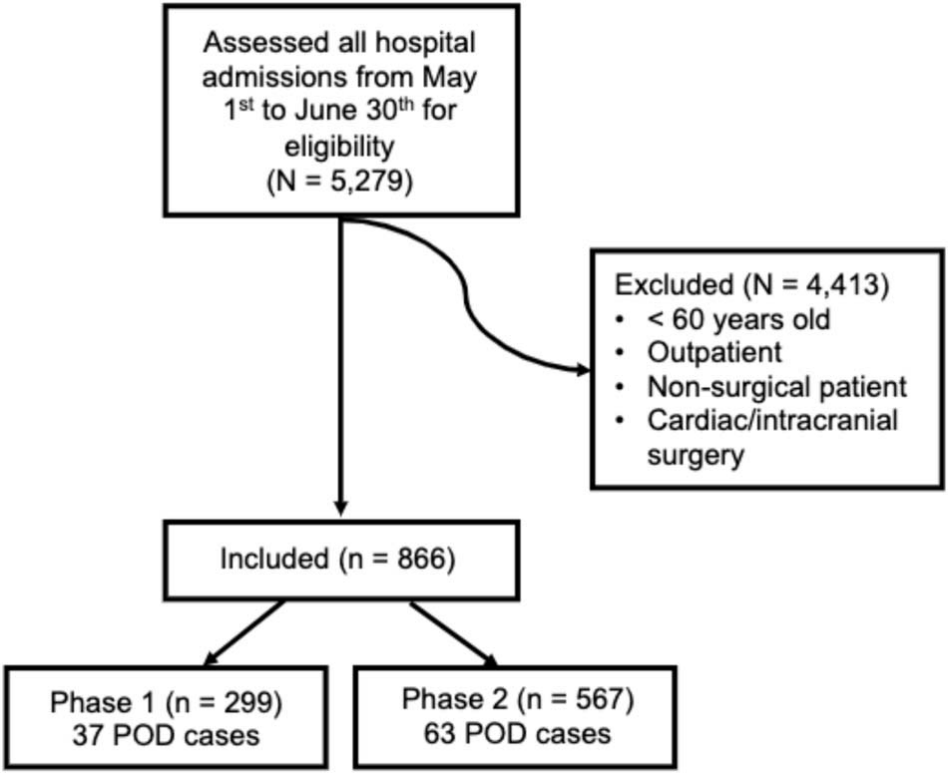
Enrolment of study participants. The flow diagram shows the total number of participants who were assessed for eligibility, those excluded and the final number of participants included in the QIP.

Figure 1 shows enrolment of subjects into the study. Baseline characteristics of the subjects were summarised by the median and first and third quartiles for numeric variables, and by frequency and percent for categorical variables, along with the percent of data missing. These summaries were stratified by phase, and differences between the phases were assessed with either a Wilcoxon test or Chi-square test, as appropriate.

Average compliance to DOSS screening was summarised graphically, using the mean [standard deviation (SD)] and median [interquartile range, (IQR)] or frequency (percent). Any difference in compliance between phases was tested using the Wilcoxon test and reported with a test statistic and *P*-value.

POD incidence was reported overall, per phase, and as the risk difference between phases with 95% Wilson confidence intervals (CIs). Expected incidence per phase was computed as the mean PIPRA score and reported per phase. Logistic regression was used to detect differences in incidence across phases. The odds ratio (OR) of POD in Phase 2 compared to Phase 1 was reported with 95% CIs. To allow for a more accurate comparison across phases, results were adjusted by patient’s baseline risk, age, self-care index [24] [SPI—‘selbstpflegeindex’, which can range from 10 (totally dependent, high need of care) to 40 points (totally independent, no need of care)] and surgery risk [25] (cardiac risk for non-cardiac surgery). These variables were considered relevant for describing case mix across phases. Incidence of POD was also illustrated graphically, with bar charts depicting expected and observed incidence.

LOS in days (continuous) and nursing time in hours (continuous) were analysed on a log scale due to their skewed distribution. Linear regression of log LOS and log nursing time per phase were used to detect differences between phases. The results (estimates with 95% CIs) were exponentiated for interpretation on the original scale (hours and days not log hours or log days). Results adjusted for delirium risk, age, self-care index and surgical risk were also reported.

LOS was further explored by PIPRA risk group: low (PIPRA <10%), intermediate (PIPRA from 10 to 19.9%), high (PIPRA from 20 to 34.9%) and very high (PIPRA >35%). A dot plot depicted the difference in log LOS per risk group, with the subject distribution per risk group. An interaction term between LOS and risk was added to the model and the phase comparisons summarised per risk group. To explore the relationship between LOS and delirium risk, we used a rolling average of 10% of patients to compare the average LOS to the average delirium risk. The analysis was then repeated for nursing time.

All analyses were performed using R Statistical Software [26] (version 4.3.2). Reporting was guided by the Strengthening the Reporting of Observational Studies in Epidemiology (STROBE) checklist.

#### Ethical considerations

We obtained a waiver (Req-2023-00307) from the Zürich Cantonal Ethics Committee for the QIP.

## Results

### Participant description

Of 5279 consecutive hospital admissions during the study period, 866 met the inclusion criteria, with 299 patients enrolled during Phase 1 and 567 in Phase 2 (Figure 1). In Phase 1 there were 178, 65, 37 and 19 patients in low, intermediate, high and very high risk groups, respectively. In Phase 2 there were 335, 122, 53 and 57 patients in these risk groups. Since, in Phase 2, staff were trained to perform prevention on medium risk and above, 232 (= 122 + 53 + 57) patients should have received preventive measures. 21.6% of eligible patients were medium risk.

Overall, the median age of participants was 72 years and 46.1% of participants were female. Only a small percentage of subjects reported, or had chart evidence of, cognitive impairment or past delirium (both 3.7%) or were undergoing highest risk procedures (surgical risk 3, 5.4%). The self-care index indicated that the study sample could generally perform self-care activities. Subject characteristics are summarised in Table 1.

**Table 1:** Description of included subjects and outcomes. Unless otherwise indicated, data shown are *n* (%-percentage of total) or median [IQR]. BMI: body mass index; SPI: ‘Selbstpflegeindex’ self-care index; CRP: C-reactive protein. ^a^Preoperative; ^b^cardiac risk for non-cardiac surgery; ^c^PIPRA risk score.

	Overall	Phase 1	Phase 2	p-value	Missing %
Number of patients	866	299	567		
Age in years [IQR]	72 [66, 78]	72 [66, 77]	72 [66, 78]	0.86	0.0
Female (%)	399 (46.1)	129 (43.1)	270 (47.6)	0.24	0.0
BMI [IQR]	25.14 [22.73, 28.01]	25.04 [22.95, 28.03]	25.17 [22.52, 28.00]	0.62	5.0
Cognitive impairment (%)	24 (3.7)	9 (4.2)	15 (3.5)	0.81	25.2
History of delirium (%)	24 (3.7)	5 (2.3)	19 (4.4)	0.27	24.5
SPI [IQR]	38 [33, 40]	38 [33, 40]	39 [34, 40]	0.77	5.0
Number of medications^a^ [IQR]	4 [2, 7]	4 [2, 6]	4 [3, 7]	0.14	21.2
log(CRP)	0.97 [−0.69, 2.81]	0.81 [−0.36, 2.47]	0.99 [−0.69, 3.05]	0.98	72.5
Surgical risk^b^ (%)				0.12	0.1
1	253 (29.2)	75 (25.1)	178 (31.4)		
2	565 (65.3)	205 (68.6)	360 (63.6)		
3	47 (5.4)	19 (6.4)	28 (4.9)		
Delirium risk^c^ [IQR]	0.07 [0.04, 0.16]	0.07 [0.04, 0.16]	0.08 [0.03, 0.16]	0.91	0.0
Outcomes:					
POD (%)	100 (11.5)	37 (12.4)	63 (11.1)		0.0
Death (%)	7 (0.8)	3 (1.0)	4 (0.7)		0.0
LOS in days:					
Mean (SD)	4.84 (4.72)	5.23 (5.32)	4.64 (4.35)		0.0
Median [IQR]	3.15 [2.01, 6.00]	3.63 [2.02, 6.14]	3.12 [2.00, 5.96]		0.0
Nursing time in hours					
Mean (SD)	23.58 (31.48)	24.39 (29.63)	23.15 (32.43)		0.1
Median [IQR]	14.73 [8.73]	15.05 [8.70, 27.39]	14.62 [8.73, 24.01]		0.1

Compliance to delirium screening fluctuated between 42% and 76.2% over time, after a substantial initial increase from study day one to study day 2 (Supplementary Figure S1). The median screening compliance was 58.8% and 64% for Phases 1 and 2, respectively, with no significant differences apparent across phases (Wilcoxon test, W = 341, *P*-value = .19).

### Delirium incidence

**Figure 2:**
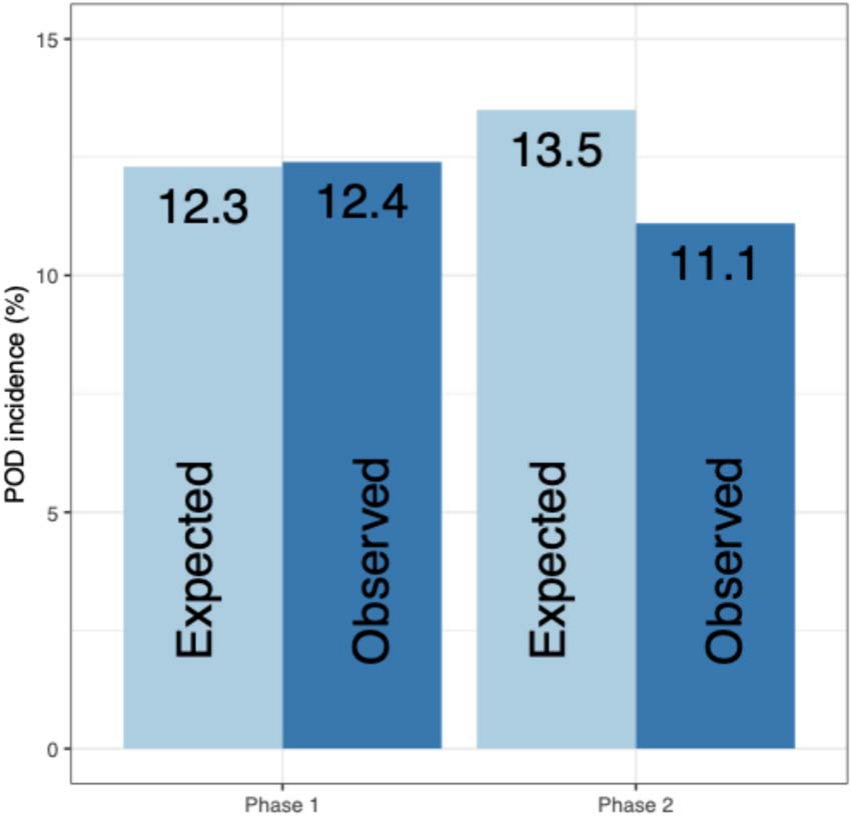
Delirium incidence across Phases 1 and 2. ‘Expected’ was the expected delirium incidence (in percent), based upon the average delirium risk (PIPRA score). ‘Observed’ was the observed delirium incidence (also in percent).

Based upon the PIPRA score, the expected incidence of POD was 12.3% and 13.5% in Phases 1 and 2, respectively. The overall observed incidence in the study was 11.5% (95% CI 9.6 to 13.8%), with incidences of 12.4% (95% CI 9.1 to 16.6%) in Phase 1 and 11.1% (95% CI 8.8 to 14.0%) in Phase 2 and a POD risk difference of −1.3% (95% CI –6.1 to 3.1%) (Figure 2). The unadjusted OR comparing the odds of POD in Phase 2 to Phase 1 was 0.89 (95% CI 0.58 to 1.37) and the adjusted OR was 0.71 (95% CI 0.44 to 1.16). These results suggest that the odds of POD in Phase 2, following introduction of PIPRA risk prediction and delirium prevention, were 29% lower than in Phase 1. However, there was not enough evidence to reject the null hypothesis of no difference between phases (Table 2).

**Table 2:** Regression-based assessment of POD risk, LOS and nursing time according to study phase. The OR (95% CIs) comparing the odds in Phase 2 to Phase 1 for being positively screened for POD and the exponentiated coefficients for linear regression of log LOS and log nursing time on phase are presented. The adjusted model takes into account the individual baseline delirium risk (PIPRA score), age, self-care index (SPI) and surgery risk.

	Unadjusted assessment (95% CIs)	*n*	Adjusted assessment (95% CIs)	*n*
POD	0.89 (0.58 to 1.37)	866	0.71 (0.44 to 1.16)	822
LOS	0.93 (0.83 to 1.04)	866	0.94 (0.85 to 1.05)	822
Nursing time	0.94 (0.84 to 1.06)	865	0.96 (0.86 to 1.7)	821

### Length of stay

The median LOS was 3.63 days in Phase 1 and 3.12 days in Phase 2 (Table 1). As LOS displayed a right-skewed distribution, it was analysed on a log scale. Phase 2 was associated with an LOS 0.93 (95% CI 0.83 to 1.04) times that of Phase 1. The multiplicative factor was 0.94 (95% CI 0.85 to 1.05), when adjusting for baseline patient characteristics. These results suggested a 6%–7% decrease in LOS in Phase 2 compared to Phase 1; however, the results were not statistically significant (Table 2).

### Nursing time

The median nursing time was 15.05 hours in Phase 1 and 14.62 hours in Phase 2 (Table 1). In Phase 2, nursing times were 0.94 (95% CI 0.84 to 1.06) times those of Phase 1. After adjusting, the multiplicative factor was 0.96 (95% CI 0.86 to 1.07). This indicated a 4%–6% reduction in nursing time in Phase 2 compared to Phase 1, although these results were not statistically significant (Table 2).

### The intervention was most effective for patients with medium risk

**Figure 3:**
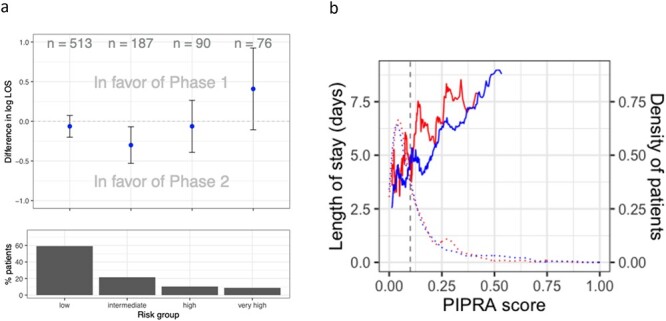
The effect of Phase 2 on LOS by delirium risk. (a) The difference in LOS (log scale) with 95% CIs (above) and percent patients (below) per risk group. (b) Average LOS versus the rolling average of delirium risk (PIPRA score) according to phase (solid lines, Phase 1 in red (above), Phase 2 in blue (below), vertical line represents threshold PIPRA score for targeted preventive measures). The density of patients is shown as a dashed line. The rolling average is for 10% of patients.

We further explored the relationship between LOS outcomes and delirium risk. We found that introduction of PIPRA risk prediction with delirium prevention was most effective for patients with a medium delirium risk (Figure 3a). In these patients, we see a statistically significant adjusted difference of −0.31 log days (95% CI from −0.53 to −0.09) in Phase 2 compared to Phase 1 (Supplementary Table S1), which is equivalent to a 26% reduction (effect estimate: 0.74, 95% CI from 0.59 to 0.92) or 1.36 days less. Whilst low and high risk patients seemed unaffected, very high risk patients showed a trend favouring no interventions (Figure 3a). To understand this, we plotted the 10% rolling average of LOS against delirium risk. The average LOS generally increased proportionally with delirium risk and the average LOS by delirium risk remained lower in Phase 2 compared to Phase 1. It is worth noting that Phase 2 had more higher risk patients than Phase 1, which may be responsible for the trend (Figure 3b).

We repeated the analysis, to explore the relationship between nursing time and delirium risk, and to understand if the intervention ultimately required more time or saved time. In medium risk patients the required nursing time was 0.24 log hours (95% CI 0.005 to 0.48) lower in Phase 2 compared to Phase 1, equivalent to a 21% reduction in hours (effect estimate: 0.79, 95% CI from 0.62 to 1.00, adjusted model; Figure S2a) or a time saving of 19.3 h per medium risk patient. We also see that Phase 2 had more patients with very high risk who required significantly more nursing time (Figure S2b).

## Discussion

### Summary

Non-pharmacological delirium prevention has been shown to reduce POD incidence by up to 54% [16] and LOS by up to 5 days [27]. However, to the best of our knowledge, none of those studies used an automated tool for the identification of at-risk patients. A novel finding from this study is the effect of delirium prevention on LOS being associated with delirium risk.

Delirium is unavoidable in some patients [28], and it is possible that those who have the highest risk may not benefit from preventive interventions; our study is the first to show that patients specifically with medium risk will indeed benefit from preventive strategies.

A cost effectiveness analysis was also performed on this study and found the cost savings for the next 10 000 eligible patients to be valued at 30 million CHF to the healthcare system, and 25 million CHF for the hospital sector only [1]. At the time of the study, these savings were equivalent to 31 million EUR and 26 million EUR, respectively.

### Strengths

Key strengths of this study were the use of real-life data and the introduction of a healthcare improvement model that truly benefits older people [29]. As the study was conducted as a QIP and all participants were consecutively enrolled, there was no selection bias introduced through a consenting process. The dataset provides an accurate representation of older patients, including those with mild cognitive impairment or dementia and low educational backgrounds, all of whom have been shown to be more at risk of delirium [3, 30]. These patient groups are frequently underrepresented in studies as they are often lost in the consenting process.

All the data was prospectively collected, with accurate measurement of LOS and nursing time, directed training of healthcare providers, and measurement of compliance to delirium screening, ensuring high data quality.

Finally, by structuring this QIP as a before–after study, we avoided the spillover effects that might hamper randomised controlled trials when improved delirium awareness in participating healthcare professionals could influence both control and intervention groups.

### Limitations

This study was designed as a QIP and not a clinical trial, which meant that we were limited in the scope of the data that we were permitted to use, in order to maintain compliance with the ethics committee waiver. It also meant that the study was not statistically powered to specifically answer all the questions we posed.

Whilst we were not able to formally structure the project as a trial, we did approach it according to best practices for a before–after study. Before–after studies carry a risk of confounder bias since the allocation into control and intervention groups is not randomised. We used the delirium risk as a pre-test to control for this bias. We also used a multivariate regression to attempt to isolate the effect of the intervention. Finally, the time period was kept short (2 months) to minimise the inherent risk of confounder bias in this type of study.

The DOSS is practical to use but is not considered the gold standard for delirium diagnosis [31]. In addition, whilst the delirium screening compliance in this study would be considered adequate for normal clinical practice, in a trial the compliance would be closer to 100%. We attempted to mitigate these factors by training the nurses to recognise and formally diagnose delirium in general, presenting the DOSS as a mandatory supportive tool with the caveat that especially hypoactive cases might be false-negatives. It is worth noting that the potential bias from the use of the DOSS should have equally affected both the control and intervention groups.

Three factors may have decreased the apparent effectiveness of the intervention. Firstly, to get an accurate assessment of delirium incidence, the staff were trained to identify and treat delirium, which may have impacted delirium incidence and LOS during Phase 1.

Secondly, there was possible contamination or delay from delirium prevention training undertaken by the nursing staff; we did attempt to mitigate this by switching on PIPRA and handing out the prevention pocket guides only in Phase 2.

Thirdly, the delirium team was already present in the hospital and the observed effectiveness may be higher in hospitals that implement all measures at once. However, whilst these three factors may have resulted in a bias, they are all in favour of the control.

## Conclusions

In this QIP, we have shown that the implementation of PIPRA, in combination with non-pharmacological multicomponent prevention, reduces LOS and nursing time in medium risk older patients. This highlights the benefits of using a validated, regulatory-grade, automated delirium risk assessment tool to identify those patients who will likely benefit most from prevention measures.

## Supplementary Material

aa-24-0848-File003_afae219

## Data Availability

Research data are not shared, but requests for collaboration are encouraged.
