# Supplementary material for: Benefits of an automated postoperative delirium risk prediction tool combined with non-pharmacological delirium prevention on delirium incidence and length of stay: a before–after analysis based on a quality improvement project
Source: Age Ageing. 2024 Oct 14;53(10):afae219. doi: 10.1093/ageing/afae219 (PMC11471309; doi:10.1093/ageing/afae219)
Supplement: aa-24-0848-File003_afae219 [file aa-24-0848-file003_afae219.docx]

**Supplementary Figure S1**: DOSS compliance by phase


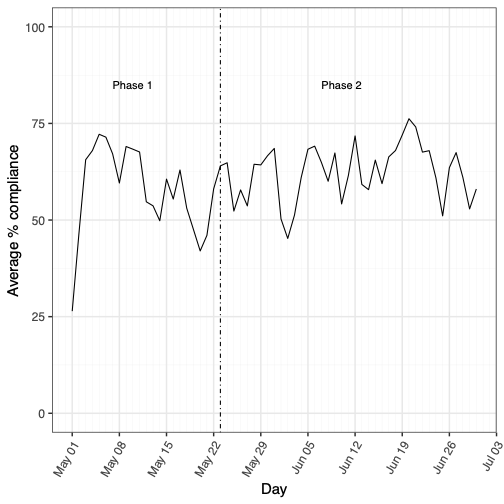


|  | **Overall** | **Phase 1** | **Phase 2** |
| --- | --- | --- | --- |
| Avg. % DOSS compliance |  |  |  |
| mean (SD) | 60.60 (9.14) | 57.54 (11.41) | 62.32 (7.17) |
| median [IQR] | 61.51 [54.16, 67.57] | 58.83 [50.63, 67.48] | 64.01 [57.95, 67.51] |

**Figure S1: DOSS compliance per phase.** The graph shows the average compliance (in percent) to DOSS delirium screening over time across all wards, with a summary table below.

**Figure S2:**

A B


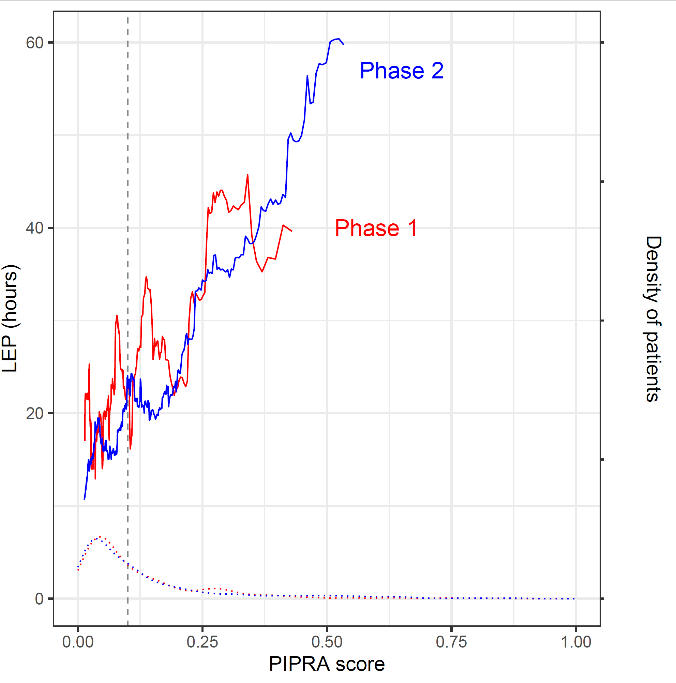


**Figure S2: The effect of phase 2 on nursing time by delirium risk.** (A) The difference in nursing time (log scale) with 95% confidence intervals (above) and percent patients (below) per risk group. The LEP assessment was not performed for one patient in phase 2 in the high risk group and was excluded from the analysis. (B) Average nursing time versus the rolling average of delirium risk (PIPRA score) according to phase (solid lines, Phase 1 in red, Phase 2 in blue, grey vertical line represents threshold PIPRA score for targeted preventive measures). The density of patients is shown as a dashed line. The rolling average is for 10% of patients.

**Supplementary Table S1:**

|  |  | Unadjusted analysis | | | | Adjusted analysis | | | |
| --- | --- | --- | --- | --- | --- | --- | --- | --- | --- |
| Outcome | **Risk group** | **Estimate (log)** | **SE** | **CI** | ***p*-value** | **Estimate (log)** | **SE** | **CI** | ***p*-value** |
| Length of stay | Low | -0.09 | 0.07 | 0.92 (0.8 to 1.05) | 0.22 | -0.02 | 0.07 | 0.98 (0.86 to 1.12) | 0.80 |
|  | Intermediate | -0.34 | 0.12 | 0.71 (0.56 to 0.9) | 0.005 | -0.31 | 0.11 | 0.74 (0.59 to 0.92) | 0.007 |
|  | High | -0.03 | 0.16 | 0.97 (0.7 to 1.33) | 0.84 | 0 | 0.15 | 1 (0.74 to 1.35) | 0.99 |
|  | Very high | 0.43 | 0.2 | 1.54 (1.04 to 2.28) | 0.032 | 0.37 | 0.19 | 1.45 (1 to 2.12) | 0.053 |
| Nursing time | Low | -0.11 | 0.08 | 0.9 (0.77 to 1.04) | 0.15 | -0.04 | 0.07 | 0.96 (0.83 to 1.10) | 0.56 |
|  | Intermediate | -0.27 | 0.13 | 0.76 (0.59 to 0.97) | 0.03 | -0.24 | 0.12 | 0.79 (0.62 to 1.00) | 0.049 |
|  | High | -0.03 | 0.17 | 0.97 (0.7 to 1.36) | 0.87 | 0.02 | 0.16 | 1.02 (0.74 to 1.41) | 0.90 |
|  | Very high | 0.64 | 0.21 | 1.9 (1.26 to 2.88) | 0.002 | 0.55 | 0.02 | 1.74 (1.17 to 2.6) | 0.007 |

**Supplementary Table S1:** Log estimates, Standard Error (SE), effect estimates with confidence intervals (CI), and p-value for the outcomes length of stay and nursing time for each risk group unadjusted and adjusted for patient characteristics.
